# Occupational Determinants of Depression Severity among Healthcare Workers: Insights from a 12-Year Study

**DOI:** 10.1192/j.eurpsy.2026.11433

**Published:** 2026-07-31

**Authors:** A. Gaddour, A. Chouhane, I. Kacem, I. Jammeli, R. Ghezal, M. Bouhoula, O. Elmaalel, A. Aloui, M. Maoua, H. Kalboussi, S. Chatti, A. Brahem

**Affiliations:** 1 Occupational Medicine Department, Ibn El Jazzar teaching Hospital, Kairouan; 2 Faculty of Medicine of Sousse, Research Laboratory LR19SP03, University of Sousse; 3 Occupational Medicine Department; 4 Occupational Medicine Department, Farhat Hached Teaching Hospital; 5 Family Medicine Department, University of Sousse; 6 Occupational Medicine Department, Sahloul Teaching Hospital; 7 Occupational Medicine Department, University of Sousse, Sousse, Tunisia

## Abstract

**Introduction:**

Healthcare workers (HCWs) are regularly exposed to high levels of occupational stress, which can contribute to the development of depressive disorders. Factors such as excessive workload, night shifts, exposure to critical situations, and frequent job changes have been linked to increased mental health risks. This study aims to analyze the relationship between work conditions and the onset and severity of depressive disorders among HCWs in Sousse, Tunisia.

**Objectives:**

To evaluate the impact of specific occupational factors on the prevalence and severity of depressive disorders among healthcare workers and to identify work-related risk factors contributing to the deterioration of their mental health

**Methods:**

A retrospective descriptive study was conducted among HCWs in Sousse, Tunisia, who took long-term sick leave due to depressive disorders between January 2010 and December 2021. Data were collected from medical records and supplemented by a questionnaire detailing workplace conditions, job roles, and psychiatric history.

**Results:**

A total of 650 cases of depression were recorded, with 81% of the affected workers being female and the majority (43%) being nurses. Workers in emergency, surgical, and critical care units accounted for 28% of the cases, with significantly higher rates of severe depression (87.3%). Night shifts were a key factor, with 91.7% of those working two or more night shifts per week experiencing severe depression. Other significant factors included job strain, a history of psychiatric illness (14.2%), and exposure to emotionally taxing events, such as patient deaths (42%).

**Conclusions:**

This study highlights the critical role of occupational factors in the development and exacerbation of depressive disorders among HCWs. Targeted interventions, such as reducing job strain, improving work-life balance, and offering mental health support, are essential to protect the mental well-being of healthcare professionals.

**Disclosure of Interest:**

None Declared

